# Peripheral-to-central extracorporeal corporeal membrane oxygenation switch in refractory cardiogenic shock patients: outcomes and bridging strategies

**DOI:** 10.1186/s13613-024-01382-3

**Published:** 2024-10-07

**Authors:** Aurélie Besnard, Quentin Moyon, Guillaume Lebreton, Pierre Demondion, Guillaume Hékimian, Juliette Chommeloux, Matthieu Petit, Melchior Gautier, Lucie Lefevre, Ouriel Saura, David Levy, Matthieu Schmidt, Pascal Leprince, Charles-Edouard Luyt, Alain Combes, Marc Pineton de Chambrun

**Affiliations:** 1 Service de Médecine Intensive-Réanimation, Sorbonne Université, Assistance Publique-Hôpitaux de Paris (AP-HP), Hôpital La Pitié–Salpêtrière, 47–83, Boulevard de l’Hôpital, Paris Cedex, 75651 France; 2 Syndrome des Anticorps Anti-phospholipides et Autres Maladies Auto-Immunes Systémiques Rares, Sorbonne Université, AP-HP, Hôpital La Pitié–Salpêtrière, Centre de Référence National Lupus Systémique, Institut E3M, Service de Médecine Interne 2, Paris, France; 3 Service de Chirurgie Cardio-Thoracique, Sorbonne Université, AP-HP, Hôpital La Pitié–Salpêtrière, Paris, France; 4 Sorbonne Université, INSERM, UMRS_1166-ICAN, Institut de Cardiométabolisme et Nutrition (ICAN), Paris, F-75013 France; 5 Centre d’Immunologie et des Maladies Infectieuses (CIMI-Paris), Sorbonne Université, Inserm, Paris, France; 6 Institut de Chirurgie Cardiaque, Hôpital Privé Jacques-Cartier, Ramsay Santé, 6 avenue du Noyer Lambert, 91300 Massy, France

**Keywords:** VA-ECMO, Central ECMO, Mechanical circulatory support, Extracorporeal membrane oxygenation, Cardiac transplantation

## Abstract

**Background:**

Peripheral veno-arterial extracorporeal membrane oxygenation (pECMO) has become the first-line device in refractory cardiogenic shock (rCS). Some pECMO complications can preclude any bridging strategies and a peripheral-to-central ECMO (cECMO) switch can be considered as a bridge-to-decision. We conducted this study to appraise the in-hospital survival and the bridging strategies in patients undergoing peripheral-to-central ECMO switch.

**Methods:**

This retrospective monocenter study included patients admitted to a ECMO-dedicated intensive care unit from February 2006 to January 2023. Patients with rCS requiring pECMO switched to cECMO were included. Patients were not included when the cECMO was the first mechanical circulatory support.

**Results:**

Eighty patients, with a median [IQR25-75] age of 44 [29–53] years at admission and a female-to-male sex ratio of 0.6 were included in the study. Refractory pulmonary edema was the main switching reason. Thirty patients (38%) were successfully bridged to: heart transplantation (*n* = 16/80, 20%), recovery (*n* = 10/80, 12%) and ventricle assist device (VAD, *n* = 4/30, 5%) while the others died on cECMO (*n* = 50/80, 62%). The most frequent complications were the need for renal replacement therapy (76%), hemothorax or tamponade (48%), need for surgical revision (34%), mediastinitis (28%), and stroke (28%). The in-hospital and one-year survival rates were 31% and 27% respectively. Myocardial infarction as the cause of the rCS was the only variable independently associated with in-hospital mortality (HR 2.5 [1.3–4.9], *p* = 0.009).

**Conclusions:**

The switch from a failing pECMO support to a cECMO as a bridge-to-decision is a possible strategy for a very selected population of young patients with a realistic chance of heart function recovery or heart transplantation. In this setting, cECMO allows patients triage preventing from wasting expensive and limited resources.

**Supplementary Information:**

The online version contains supplementary material available at 10.1186/s13613-024-01382-3.

## Introduction

Short-term mechanical circulatory support (MCS) drastically improved the outcome of refractory cardiogenic shock [1]. Peripheral veno-arterial extracorporeal membrane oxygenation (pECMO) has become the first-line device in this setting since it provides both respiratory and cardiac supports, is easy to insert, even at the bedside, and provides stable flow rates [2]. Patients with cardiac function recovery will be weaned, while the others can be bridged to transplantation or long-term MCS. Some pECMO complications (i.e. refractory pulmonary edema, cannulation site cellulitis) can preclude any bridging strategies and a peripheral-to-central ECMO (cECMO) switch can be considered as a bridge-to-decision. The outcome of cECMO has been extensively reported in post-cardiotomy cardiogenic shock with many studies reporting > 500 patients with cECMO [3, 4]. On the contrary, data on medical patients with pECMO upgraded to cECMO are scarce and we believe this needs further investigation [5].

We conducted this study to appraise the in-hospital survival and the bridging strategies in refractory cardiogenic shock patients undergoing peripheral-to-central MCS switch.

## Materials and methods

### Patients

We conducted a retrospective monocenter study including medical patients admitted to our 26-bed ECMO-dedicated intensive care unit (ICU) from February 2006 to January 2023. Patients were included when meeting the following criteria: medical refractory cardiogenic shock requiring pECMO and thereafter switched to cECMO. Patients were not included when the cECMO was the first MCS (postcardiotomy patients). These inclusion criteria were designed to include only medical patients requiring pECMO who were subsequently switched to cECMO.

### Reason for peripheral-to-central ECMO switch

There was no pre-specified protocol in our center for peripheral-to-central ECMO switch indications. Decisions were taken on a case-to-case basis by a multidisciplinary team including cardiologists, intensive care medicine physicians, cardiac surgeons, and cardiac transplantation specialists. The main reasons leading to centralization were: refractory pulmonary edema; infectious or vascular peripheral complication of the pECMO; left heart cavities pre-thrombotic state and refractory circulatory failure responsible for multiple organ failure precluding any chance of short-term bridge to transplantation or long-term MCS. Centralization for pulmonary edema was considered as a last resort after unsuccessful or impractical left ventricle unloading using an intra-aortic balloon pump, IMPELLA^®^, or atrial septostomy. Centralization for infectious or peripheral vascular complications was considered as a last resort after an unsuccessful or impractical switch to a femoral vein-to-axillary artery ECMO.

### Cannulation sites

Peripheral cannulation was defined as the implantation of the ECMO arterial line in the femoral or axillary artery. Central cannulation was defined by at least one ECMO arterial line directly implanted in the ascending aorta through surgical thoracotomy. Central cannulation was thereafter categorized into three groups, according to the number of cannulas: 2 cannulas group (right atrium to ascending aorta), 3 cannulas group (right atrium to ascending aorta with an additional venting cannula in the superior left pulmonary vein, the pulmonary artery or the apex of the left ventricle) and 4 cannulas group (bicentrifugal biventricular support from the right atrium to the pulmonary artery and from the left ventricle apex to the ascending aorta). Several factors at our center influenced the choice of cECMO configuration, with the main factor being the improvements in surgical technique. Initially, cECMO consisted of a two-cannula setting. Subsequently, a drainage cannula was added to reduce pulmonary edema. Eventually, a four-cannula setting was considered the highest standard of care as it provided transpulmonary flow.

### Data collection

The following information was collected in standardized forms: epidemiological parameters; acute heart failure clinical, biological, and therapeutic history; clinical manifestations; laboratory findings on centralization day; reason for centralization; complication(s) of the procedure; in-ICU organ-support treatments including mechanical ventilation and dialysis; MCS-weaning status; bridge-to-recovery, transplantation or ventricular assist device (VAD); ICU complications; vital, transplantation and long-term circulatory support status at ICU and hospital discharges and at last follow-up.

### Outcome measures

The primary endpoint was in-hospital survival, defined as the proportion of patients discharged alive from the hospital. The secondary endpoints included: the proportion of patients successfully bridged (to recovery, transplantation, or long-term MCS) and the one-year survival rate.

### Statistical analysis

We followed the STROBE (Strengthening the Reporting of Observational Studies in Epidemiology) recommendations for reporting cohort studies. All consecutive adult patients who presented refractory cardiogenic shock requiring pECMO and thereafter switched to cECMO during the study period were included. No sample size calculation was performed. Patient characteristics were expressed as numbers (percentages) for categorical variables, and median (interquartile range (IQR)) for continuous variables. Categorical variables were compared by chi-square or Fisher’s exact test, whereas continuous variables were compared by Student’s or Wilcoxon’s rank-sum tests. After comparing patients based on the primary endpoint, we conducted a subgroup analysis in patients who received bi-centrifugal circulatory support. Additionally, we compared patient outcomes across three distinct study periods: 2006–2011, 2012–2017, and 2018–2023. Kaplan-Meier overall survival curves until Day 360 were computed and compared using Log-rank tests. Baseline risk factors of death at hospital discharge were assessed using a multivariate Cox proportional hazards model. Baseline variables (i.e. obtained before cECMO start) included in the multivariable model were defined a priori, and no variable selection was performed. Variables considered for regression analysis and corresponding number of missing values are provided in Supplemental Table 1. Multiple imputations were used to replace missing values when appropriate. Ten copies of the dataset were created with the missing values replaced by imputed values, based on observed data including outcomes and baseline characteristics of participants. Each dataset was then analyzed and the results from each dataset were pooled using Rubin’s rule. Hazard ratios and their 95% confidence interval were estimated. Statistical significance was set at *p* < 0.05. All analyses were conducted using R version 4.2.1.

### Ethical considerations

The database is registered with the “*Commission Nationale de l’Informatique et des Libertés*” (2217847v0). The study was conducted in accordance with the French MR004 methodology for medical research. In accordance with the ethical standards of our hospital’s institutional review board, the Committee for the Protection of Human Subjects, and French law, written informed consent was not needed for demographic, physiological and hospital-outcome data analyses because this observational study did not modify existing diagnostic or therapeutic strategies; however, patients were informed of their inclusion in the study.

## Results

### Characteristics, in-ICU organ failure and main outcomes

**Fig. 1:**
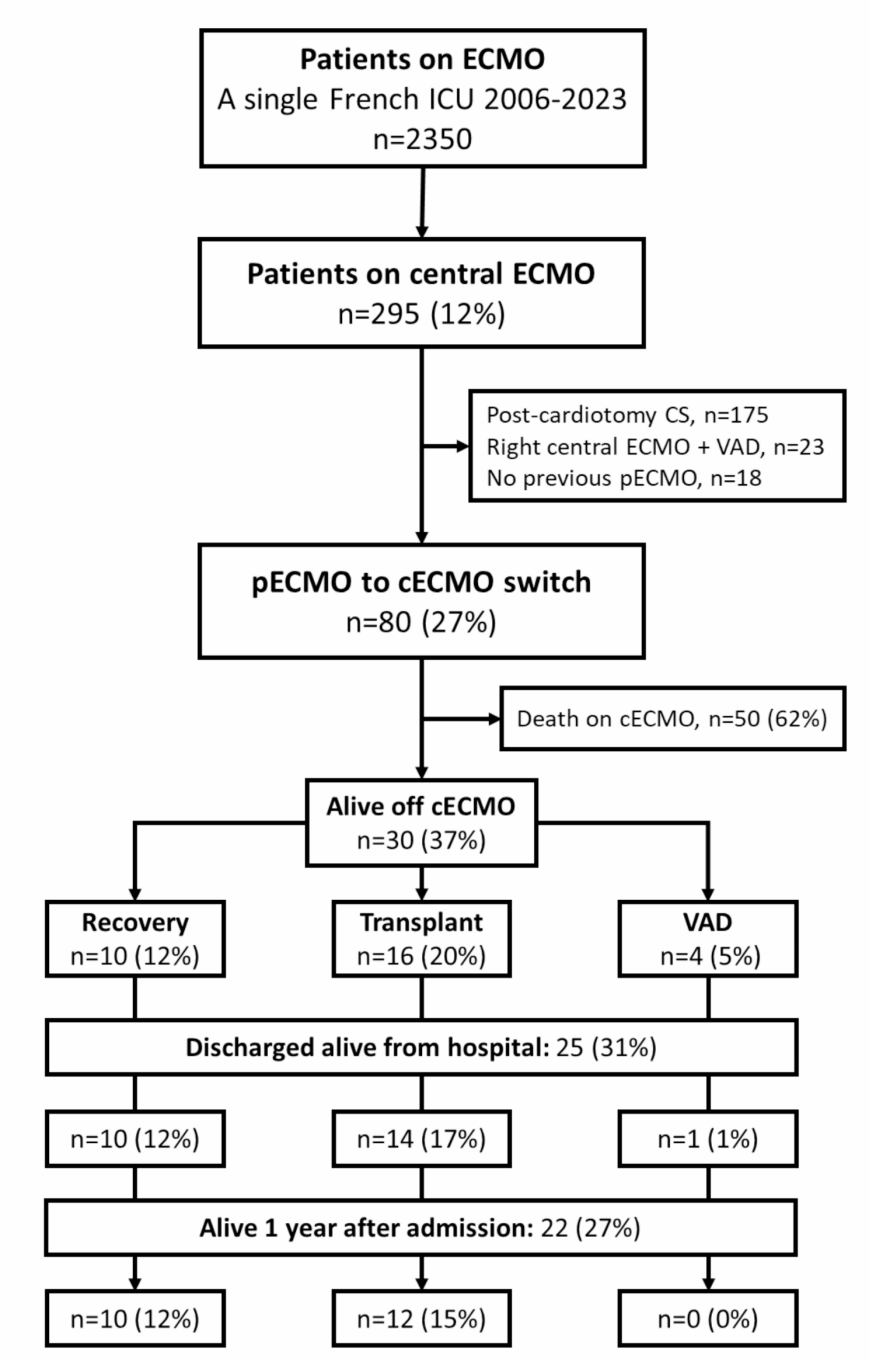
Study flow chart Abbreviations: cECMO: central extracorporeal membrane oxygenation; ICU: intensive care unit; CS: cardiogenic shock; VAD: ventricle assist device; pECMO: peripheral veno-arterial extracorporeal membrane oxygenation

**Fig. 2:**
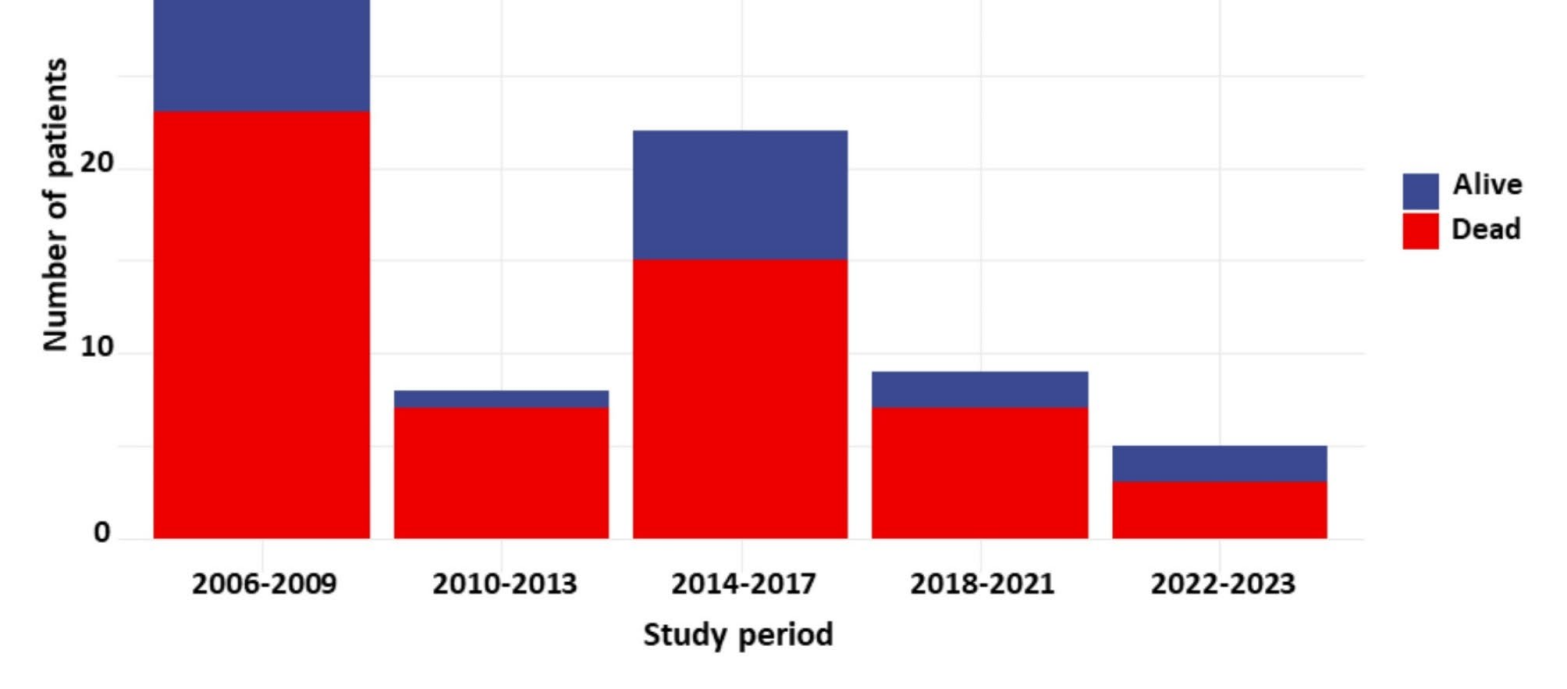
Timing repartition of cECMO during the study period and corresponding number of survivors

From February 2006 to January 2023, 295 patients had cECMO in our ICU (Fig. 1). Eighty patients, with a median age at admission 44 [29–53] years and 38%female, met the inclusion criteria and were recruited in the study (Table 1). Median ICU admission SAPS-II score was 63 [47–71]. Causes for cardiogenic shock were: myocardial infarction 38%, myocarditis 31%, dilated cardiomyopathy 24% and others 7%. 31% patients had a cardiac arrest before the first ECMO implantation. All patients had pECMO before cECMO, associated with intra-aortic balloon pump or IMPELLA in 41% and 10% of cases respectively. The median time from the first MCS to cECMO was 5 [2-15]  days. Before cECMO, 70% patients were on mechanical ventilation for more than 48 h and 54% were under renal replacement therapy. Fifty (62%) patients died on cECMO, 16 (20%) could be bridged-to-transplant, 10 (12%) to recovery and 4 (6%) to VAD. Survival to hospital discharge and after one year were 31% and 27%, respectively. The causes of the death on cECMO (*n* = 50) were: neurological complication 36% (stroke 20%, anoxic encephalopathy 16%), septic shock 30%, multiple organ failure 18%, hemorrhagic shock 8% and cECMO clotting 8%. The timing repartition of cECMO during the study period and the corresponding number of survivors are reported in Fig. 2. At last follow up, 18/22 (82%) patients were still alive.

**Table 1:** General characteristics before switch from pECMO to cECMO

Variables	All patients *n* = 80	Hospital discharge			*p*-value
		Survivors *n* = 25		Non-survivors *n* = 55	
Female	30 (38)	10 (40)		20 (36)	0.8
Age, years	41 ± 14	39 ± 13		42 ± 14	0.4
Body mass index, kg/m^2^	25.9 ± 5.4	24.4 ± 5.6		26.6 ± 5.2	0.08
ICU admission SAPS II score	63 [47–71]	61 [41–71]		64 [49–71]	0.4
ICU admission SOFA score	12 [8–14]	10 [8–14]		12 [8–14]	0.4
Charlson comorbidity index	1 [1–2]	1 [0.5–1.5]		2 [1–2]	0.2
Cause of the cardiogenic shock					0.004
Myocardial infarction	30 (38)	4 (16)		26 (47)	
Myocarditis	25 (31)	10 (40)		15 (27)	
Dilated cardiomyopathy	19 (24)	6 (24)		13 (24)	
Others^a^	6 (7)	5 (20)		1 (2)	
First MCS					
Cardiac arrest before first MCS	25 (31)	8 (32)		17 (31)	0.9
Peripheral ECMO	80 (100)	25 (100)		55 (100)	
IMPELLA^®^	8 (10)	2 (8)		6 (11)	> 0.99
Intraaortic balloon pump	33 (41)	7 (28)		26 (47)	0.1
Organ failures before cECMO					
Renal replacement therapy	43 (54)	9 (36)		34 (62)	0.03
Mechanical ventilation > 48 h	56 (70)	15 (60)		41 (75)	0.2
Time on MV, days	3 [1–11]	2 [1–5]		4 [2–13]	0.04
Outcomes					
Time from ICU admission to cECMO, days	8 [2–20]	8 [2–18]	8 [2–20]		0.9
Time from first MCS to cECMO, days	5 [2–15]	5 [1–13]	6 [2–18]		0.3
Time on MCS, days	30 [12–52]	27 [12–58]	30 [12–50]		0.6
Time in ICU, days	38 [18–63]	50 [35–79]	32 [13–52]		0.005
Time in hospital, days	43 [24–70]	73 [45–105]	34 [16–53]		< 0.001
Bridging strategies, *n* = 30					0.005
Bridge-to-recovery	10 (33)	10 (40)	0 (0)		
Bridge-to-transplantation	16 (53)	14 (56)	2 (4)		
Bridge-to-VAD	4 (13)	1 (4)	3 (6)		
LVAD	2 (3)	0 (0)	2 (4)		
BIVAD	1 (1)	0 (0)	1 (2)		
TAH	1 (1)	1 (4)	0 (0)		
Successful cECMO weaning	31 (39)	25 (100)	6 (11)		< 0.001
Day-90 mortality	52 (65)	0 (0)	52 (95)		< 0.001
In-hospital mortality	55 (69)	0 (0)	55 (100)		< 0.001
One-year mortality	58 (72)	3 (12)	55 (100)		< 0.001

Continuous variables are expressed as mean ± standard deviation or median [interquartile range 25–75] and compared with Student’s t-test or Wilcoxon’s rank test; categorical variables are expressed as n (%) and compared with Fischer’s exact test

Abbreviations: BIVAD: Biventricular Assist Device; cECMO: central Extracorporeal Membrane Oxygenation; ICU: Intensive Care Unit; LVAD: Left Ventricular Assist Device; MCS: Mechanical Circulatory Support; MV: Mechanical Ventilation; SAPS-II: simplified acute physiology score II; SOFA: Sequential Organ Failure Assessment; TAH: Total Artificial Heart; ECMO: Extracorporeal Membrane of Oxygenation; VAD: Ventricular Assist Device

^a^Hypertrophic cardiomyopathies *n* = 2, catastrophic antiphospholipid syndrome *n* = 1, anaphylactic shock *n* = 1, arrhythmic storm *n* = 1, iatrogenic rupture of tricuspid papillary muscle *n* = 1 and cardiogenic shock of unknown aetiology *n* = 1

### Reason for cECMO switch, complications

Reasons for cECMO switch were: refractory pulmonary oedema 61%, refractory circulatory failure 39%, cannulation site infection 22%, left heart cavities pre-thrombotic state 18% and lower limb ischemia 6% (Table 2). Four cannulas (biventricular support) were used in 67% of patients whereas three or two cannulas were used in 29% and 4% of patients, respectively. On cECMO implantation day, the median SOFA score was 17 [15–19]. The median duration of central assistance was 30 [12–52] days. Main complications on cECMO were: need for renal replacement therapy 76%, hemothorax or tamponade 48%, need for surgical revision 34%, mediastinitis 28% and stroke 28% (84% of which were fatal). Thirty-one (39%) patients could ultimately be weaned from cECMO. The timely distribution of the number of cannulas and the reasons for switching to cECMO are detailed in Supplemental Figs. 1 and 2.

**Table 2:** cECMO-Related characteristics and complications

Variables	All patient *n* = 80	Hospital discharge		*p*-value
		Survivors *n* = 25	Non-survivors *n* = 55	
Reason for cECMO switch				
> 1 reason	39 (49)	12 (48)	27 (49)	0.9
Pulmonary edema	49 (61)	15 (60)	34 (62)	0.9
Circulatory failure	31 (39)	11 (44)	20 (36)	0.5
Cannulation site infection	14 (18)	3 (12)	11 (20)	0.5
Lower limb ischemia	5 (6)	1 (4)	4 (7)	> 0.99
Cardiac cavities pre-thrombotic state	14 (18)	3 (12)	11 (20)	0.5
cECMO configuration^a^				0.7
2 cannulas	3 (4)	0 (0)	3 (6)	
3 cannulas	22 (29)	7 (32)	15 (28)	
4 cannulas	51 (67)	15 (68)	36 (67)	
Parameters on cECMO-day				
SOFA score	17 [15–19]	16 [15–18]	18 [15–20]	0.05
Platelets, G/L	99 [59–143]	117 [84–136]	87 [57–152]	0.3
Fibrinogen, g/L	4.6 [2.8–6.1]	5.1 [3.5–6.2]	4.3 [2.4–6.1]	0.3
Prothrombin time, %	56 [46–68]	60 [48–68]	55 [44–65]	0.4
Bilirubin, UI/L	26 [16–56]	22 [11–50]	34 [17–56]	0.1
Complication under cECMO				
Renal replacement therapy	61 (76)	14 (56)	47 (85)	0.004
Hemothorax or tamponade	38 (48)	9 (36)	29 (53)	0.2
Surgical revision	27 (34)	8 (32)	19 (35)	0.8
Time from cECMO to first revision, days	3 [1–7]	1 [0–2]	7 [1–14]	
Revision for bleeding	18 (22)	7 (28)	11 (20)	
Revision for mediastinitis	7 (9)	1 (4)	6 (11)	
Revision for device failure	5 (6)	2 (8)	3 (5)	
Mediastinitis	22 (28)	9 (36)	13 (24)	0.2
Time from cECMO to mediastinitis, days	17 [10–30]	28 [30–46]	13 [10–20]	
Gram negative bacteria	10 (12)	4 (16)	6 (11)	
Gram positive bacteria	9 (11)	5 (20)	4 (7)	
Fungi	9 (11)	2 (8)	7 (13)	
Stroke	22 (28)	4 (16)	18 (33)	0.1
Ischemic	12 (15)	4 (16)	8 (15)	
Haemorrhagic	5 (6)	0 (0)	5 (9)	
cECMO thrombosis	4 (5)	1 (4)	3 (5)	> 0.99
Time on cECMO, days	30 [12–52]	27 [12–58]	30 [12–50]	0.6
Successful cECMO weaning	31 (39)	25 (100)	6 (11)	< 0.001

Continuous variables are expressed as mean (standard deviation) or median [interquartile range 25–75] and compared with Student’s t-test or Wilcoxon’s rank test; categorical variables are expressed as n (%) and compared with Fischer’s exact test. Abbreviations: cECMO: central Extracorporeal Membrane Oxygenation; MCS: Mechanical Circulatory Support; SOFA: Sequential Organ Failure Assessment

^a^The exact configuration was unknown in 5 patients

### Factors associated with in-hospital mortality

**Fig. 3:**
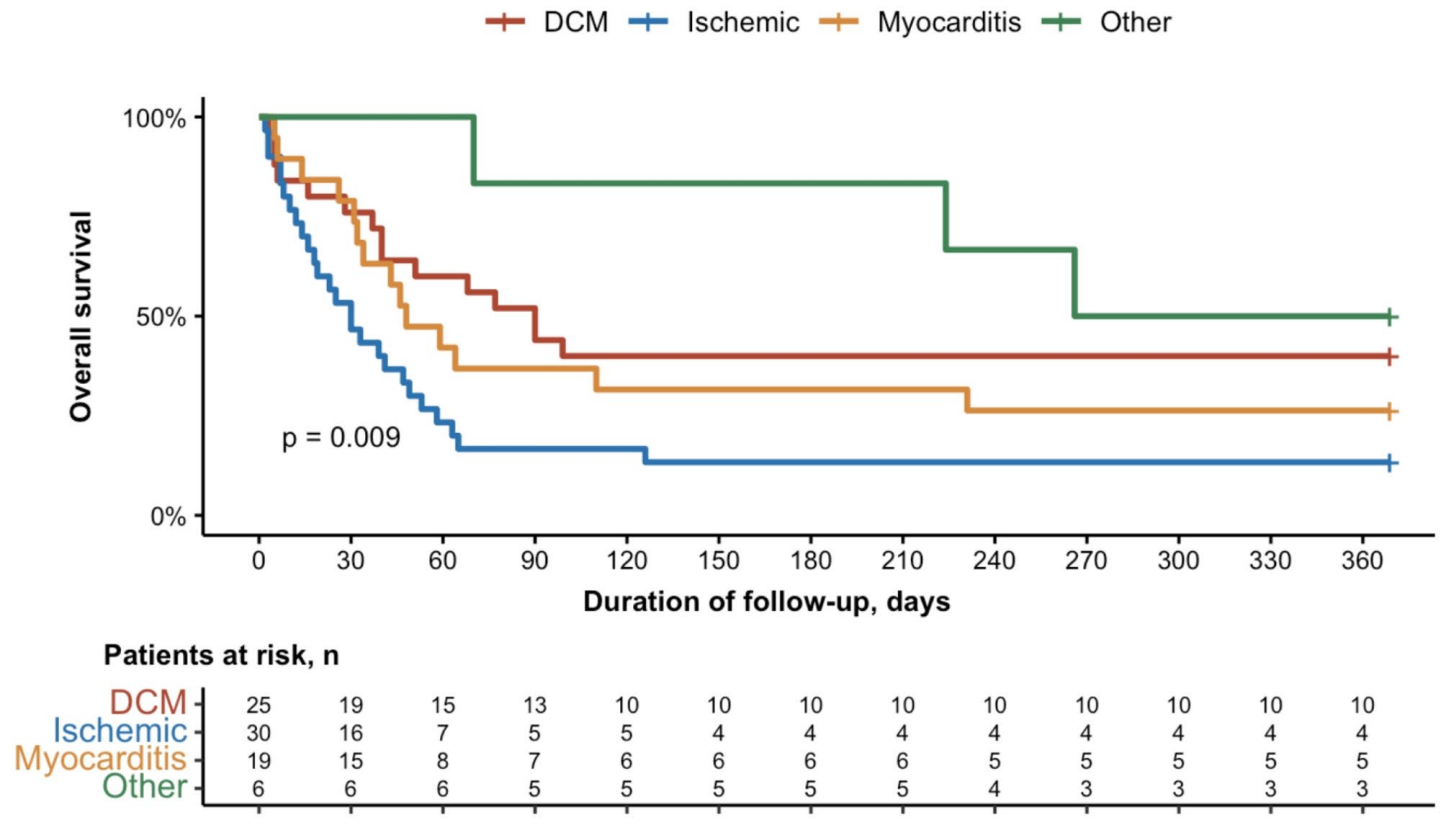
Kaplan Meier Estimates of Survival According on Cardiogenic Shock Cause Abbreviations: DCM; Dilated Cardiomayopathy. Others: hypertrophic cardiomyopathies *n* = 2, catastrophic antiphospholipid syndrome *n* = 1, anaphylactic shock *n* = 1, arrhythmic storm *n* = 1, iatrogenic rupture of tricuspid papillary muscle *n* = 1 and cardiogenic shock of unknown aetiology *n* = 1

The univariate and multivariate Cox model analysis of factors associated with in-hospital mortality are reported in Table 3. Factors significantly associated with the main endpoint in univariable analysis were: myocardial infarction as the cause of cardiogenic shock (HR 2.4 [1.2–4.5], *p* = 0.008); pre-cECMO renal replacement therapy (HR 1.9 [1.1–3.2], *p* = 0.02), the duration of MV pre-cECMO (HR 0.99 [0.9-1.0], *p* = 0.05) and SOFA score on cECMO day (1.1 [HR 1.01–1.2], *p* = 0.03). The only variable remaining associated with in-hospital mortality in the multivariable analysis was myocardial infarction as the cause of cardiogenic shock: HR 2.5 [1.3–4.9], *p* = 0.009. The Kaplan Meier estimates of survival according to the cause of the cardiogenic shock is reported in Fig. 3. An univariate and multivariate Cox model analysis of factors associated with one-year mortality are reported in Supplemental Table 2.

**Table 3:** Univariate and Multivariate Cox Model Analysis of Pre-cECMO In-Hospital Mortality-Associated factors

Variables	Univariate		Multivariate	
	HR (95% CI)	*p*-value	HR (95% CI)	*p*-value
Female	0.78 [0.45 to 1.34]	0.4		
Age, years	1.01 [0.99 to 1.03]	0.2		
Body mass index, kg/m2	1.04 [1.00 to 1.09]	0.07		
Study period				
2006–2009	—			
2010–2013	1.59 [0.68 to 3.72]	0.3		
2014–2017	1.02 [0.54 to 1.93]	0.9		
2018–2021	1.20 [0.52 to 2.80]	0.7		
2022–2023	0.82 [0.25 to 2.73]	0.7		
ICU admission SAPS II score	1.01 [1.00 to 1.03]	0.1		
ICU admission SOFA score	1.04 [0.98 to 1.10]	0.2		
Charlson comorbidity index	1.18 [0.94 to 1.48]	0.1		
Past medical history				
Arterial hypertension	1.16 [0.61 to 2.19]	0.6		
Cardiomyopathy	1.00 [0.53 to 1.90]	0.9		
Diabetes	1.32 [0.52 to 3.31]	0.6		
Immunocompromised	0.82 [0.33 to 2.06]	0.7		
Cause of the cardiogenic shock				
Myocarditis	—			
Myocardial infarction	2.38 [1.25 to 4.54]	0.008	2.50 [1.26 to 4.95]	0.009
Dilated cardiomyopathy	1.38 [0.66 to 2.86]	0.4	1.61 [0.75 to 3.43]	0.2
Others^a^	0.38 [0.09 to 1.67]	0.2	0.29 [0.04 to 2.26]	0.2
First MCS				
Cardiac arrest before first MCS	1.01 [0.57 to 1.77]	0.9		
IMPELLA^®^	1.30 [0.56 to 3.05]	0.5		
Intraaortic conterpulsation	1.50 [0.89 to 2.53]	0.1		
Organ failures before cECMO				
Renal replacement therapy	1.87 [1.09 to 3.19]	0.02	1.34 [0.58 to 3.10]	0.5
Mechanical ventilation > 48 h	1.23 [0.68 to 2.23]	0.5		
Time on MV, days	0.99 [0.98 to 1.00]	0.05		
cECMO configuration				
≤ 3 cannulas	—			
4 cannulas	0.94 [0.54 to 1.64]	0.8		
Reason for cECMO switch				
> 1 reason	1.02 [0.61 to 1.72]	0.9		
Pulmonary edema	0.99 [0.58 to 1.69]	0.9		
Circulatory failure	1.00 [0.58 to 1.73]	> 0.99		
Cannulation site infection	1.00 [0.52 to 1.94]	> 0.99		
Lower limb ischemia	1.80 [0.65 to 4.99]	0.3		
Cardiac cavities pre-thrombotic state	1.24 [0.64 to 2.39]	0.5		
cECMO-day parameters				
SOFA score	1.10 [1.01 to 1.20]	0.03	1.04 [0.91 to 1.19]	0.5
Platelets, G/L	1.00 [0.99 to 1.00]	0.6		
Fibrinogen, g/L	0.89 [0.77 to 1.02]	0.08		
Prothrombin time, %	0.99 [0.97 to 1.00]	0.09		
Bilirubin, UI/L	1.00 [1.00 to 1.00]	0.9		

Abbreviations: HR: Hazard Ratio; CI: Confidence Interval; BIVAD: Bi Ventricular Assist Device; cECMO: central Extracorporeal Membrane Oxygenation; ICU: Intensive Care Unit; MCS: Mechanical Circulatory Support; MV: Mechanical Ventilation: SAPS-II: simplified acute physiology score II; SOFA: Sequential Organ Failure Assessment

^a^Others: hypertrophic cardiomyopathies *n* = 2, catastrophic antiphospholipid syndrome *n* = 1, anaphylactic shock *n* = 1, arrhythmic storm *n* = 1, iatrogenic rupture of tricuspid papillary muscle *n* = 1 and cardiogenic shock of unknown aetiology *n* = 1

### Subgroup analysis in patients with bicentrifugal biventricular support

**Fig. 4:**
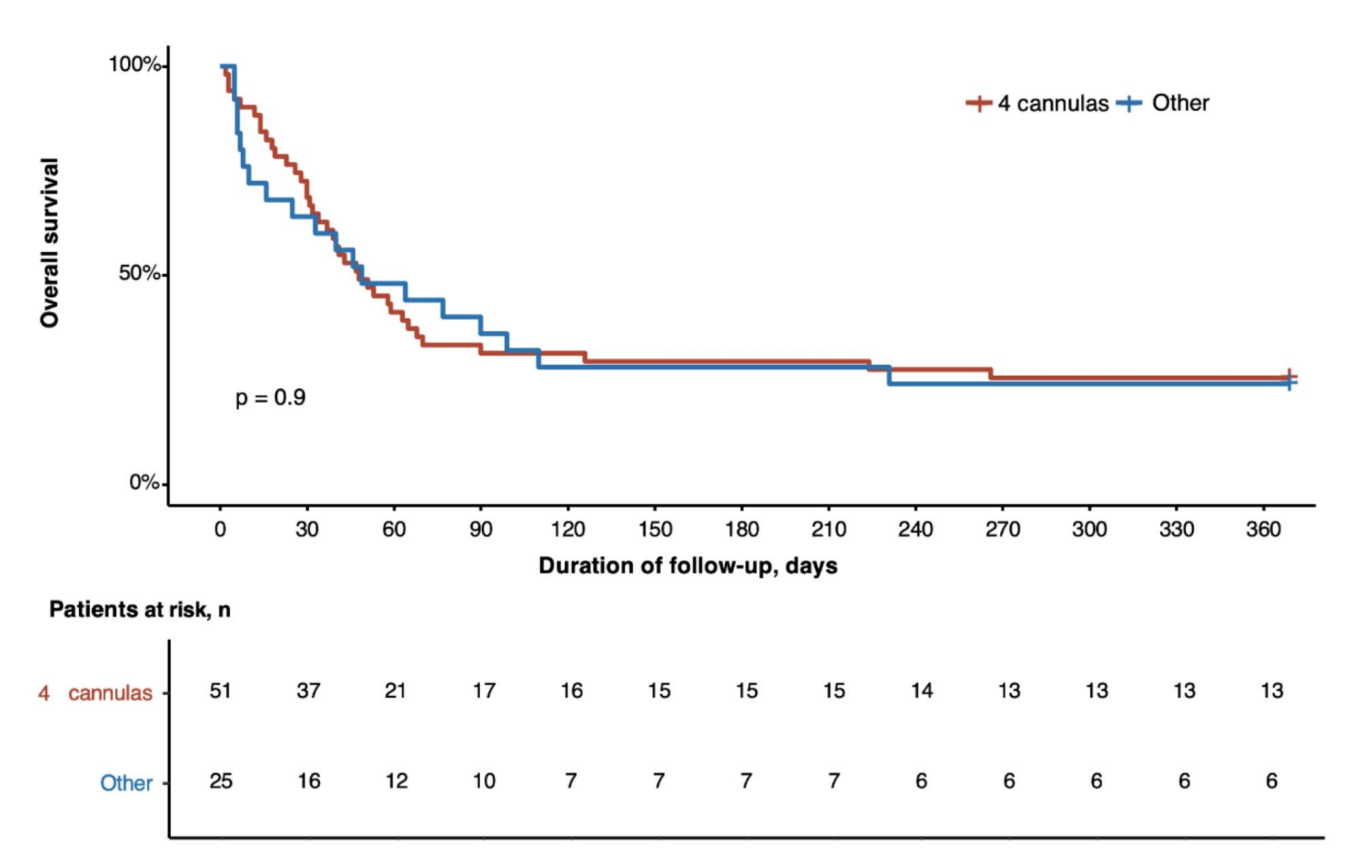
Kaplan Meier Estimates of Survival According cECMO Configuration Abbreviations: cECMO central extracorporeal membrane oxygenation

Fifty-one patients had a bicentrifugal biventricular support with a 4 cannulas cECMO configuration (Supplemental Table 3). In this subgroup, 15 (29%) patients were alive at hospital discharge, 67% bridged-to-transplantion, 4 to-recovery and 1 to-Total Artificial Heart. The Kaplan Meier estimates of survival was not statistically different between patients with bicentrifugal and those with monocentrifugal support (Fig. 4). The univariate Cox model analysis found no factors statistically significantly associated with in-hospital mortality.

### Subgroup analysis according study periods

A comparison of key variables across the three study periods is presented in Supplemental Table 4. Briefly, over the course of the study, patients who were switched from pECMO to cECMO were younger and more frequently received intra-aortic counterpulsation before cECMO. The reasons for switching, causes of cardiogenic shock, and mortality rates did not differ significantly across the study periods.

## Discussion

In our institution, peripheral ECMO is the long-standing first line MCS for any patients with refractory cardiogenic shock. Switching a patient from peripheral to central ECMO is a delicate and difficult decision. In young patients with failing pECMO support and a clinical status precluding immediate bridge to VAD or transplantation, a switch to cECMO can provide additional time until organs recover, or neurological evaluation is possible while waiting to tailor for the most appropriate bridging trajectory. Yet, cECMO is a major surgery with a high burden of complications and its outcome in this setting has been poorly investigated.

Several conclusions can be drawn from the results of our series. First, the switch from pECMO to cECMO appears limited to a very specific group of young patients with a realistic recovery or bridging project. These results are not applicable to all patients with cardiogenic shock on pECMO. Second, this is a very sick population with a very high mortality rate (27% one-year survivors). In this setting cECMO probably allows patients triage and to avoid wasting expensive and limited resources (transplant/VAD). Third, cECMO is associated with a high burden of complications including bleeding, mediastinitis, and stroke. The higher rate of stroke in patients with cECMO compared to pECMO has been previously reported and might be related to the patient’s severity, increased need for anticoagulation, cardiotomy, or anterograde flow [6, 7]. Fourth, more than half of one-year survivors had been bridged-to-transplantation, as in France, patients under cECMO can be prioritized on cardiac transplantion list. This switching strategy therefore seems to best fit heart transplantation candidates or when a cardiac recovery can be expected (i.e. myocarditis). Fifth, when adopting this strategy, one should be ready to give the patient time to recover as the median duration on cECLS in survivors was around a month and a quarter were on cECLS more than 50 days. Last, cardiogenic shock following myocardial infarction was associated with the poorest outcome, probably because this subpopulation was less likely to be a candidate for recovery or heart transplant. The use of cECMO in this indication appears to be futile.

Comparing our cohort to available literature is uneasy as very few series investigated this specific population. Previous studies investigated the outcome either of cECMO for the management of post-cardiotomy cardiogenic shock or the switch from pECMO to central extracorporeal life support (cECLS) including a small proportion of cECMO. In a large multicenter international study reporting the outcome of post-cardiotomy cardiogenic shock, 245 patients with cECMO were compared to 536 pECMO. The in-hospital mortality of cECMO was 72% with very few patients being bridged to VAD or transplant, mostly related to their higher median age (61.5 ± 14.1 years) [4]. The large ELSO series on post-cardiotomy cardiogenic shock showed that 46% of the 7185 reported patients were cannulated with cECMO, which was associated with poorer outcomes after propensity score matching [3]. In a study from Japan, including 70 patients with fulminant myocarditis under pECMO over 15 years, 69% were converted to cECLS with a 5-year survival of 71%. However, only 3 patients were switched to a 3 ECMO cannula configuration as most patients directly received a VAD [8]. In another Japanese series, 6/24 patients were bridged to a central ECMO with a favorable outcome in 5/6 cases (recovery *n* = 3, VAD *n* = 2, death *n* = 1) [9]. In both series, the population had a similar median age compared to our patients, they were very severe but had a larger predominance of fulminant myocarditis. More recently a German study reported the outcome of 58 patients receiving cECLS mostly following cardiac arrest (72%). 71% of patients were given cECLS after failing pECMO of which only 62% had a cECMO configuration (with 2 cannulas). Their mean age upon admission was 54 years and half had acute coronary syndrome. The 6-month mortality rate of patients converted from pECMO to cECMO was 79%, 4 patients received a VAD, and none were bridged to heart transplantation. The specific outcome of patients who were switched from pECMO to cECMO (and not cECLS) was not reported [10].

The main reason for pECMO switch in our series was refractory pulmonary edema. Several techniques are available to unload the left ventricle under pECMO: IABP, IMPELLA^®^, atrial septostomy and ultimately cECMO [11, 12]. IABP and IMPELLA^®^ were used in our patients, and cECMO was only considered after these first-line strategies had failed in some patients. Atrial septostomy was not routinely performed in our center during the time of the study while IMPELLA^®^ was not available in its early period (cECMO was therefore the sole venting rescuing strategy in refractory pulmonary edema). Noteworthy, some patients with pulmonary edema had also other indications for cECMO (especially peripheral complication) precluding other venting strategies. Nowadays, innovative surgical techniques using hybrid central and peripheral cannulation could be an alternative to cECMO (for instance veno-pulmonary artery ECMO using a ProtekDuo^®^ cannula associated to IMPELLA^®^ [13], biPELLA [14, 15] or left ventricle-to-axillary artery cannulation) and needs to be further investigated.

There is a trend of decreased peripheral-to-central switches over the course of the study, which might be explained by improved management of pECMO complications, more frequent use of IABP or IMPELLA for left ventricular unloading, or earlier bridge-to-transplant/VAD. For instance, 26%, 42% and 86% of patients received IABP respectively before 2012, between 2012 and 2017, and after 2017.

Our study has several strengths and limitations. It has a retrospective, monocentric, observational design but few data exist on this very specific population. Due to the inclusion period, a heterogeneity of management may have occurred. Specifically, as the cECMO cannulation techniques have changed during the span of the study, we included the number of cannulas in the analysis without disclosing any impact in terms of prognosis. The was no pre-specified protocol for cECMO switch as there are no guidelines for ECMO centralization. Decisions such as the initiation of cECMO, the timing of the switch, and the specific cECMO configuration may reflect our institution’s practices and clinical experience. This might have induced a selection bias; however, this study reflects real life, in which centralization decisions are taken after a multidisciplinary cross-talk between intensivists, cardiac transplant physicians, and cardiac surgeons. We did not perform any comparative analysis with a control group of pECMO patients who were not switched to cECMO. Yet, we believe it has only minimal interest given the extremely selected nature of this population.

## Conclusion

The switch of failing pECMO support to a cECMO as a bridge-to-decision is a possible strategy for a very selected population of young patients with a realistic chance of recovery or heart transplantation. cECMO allows patient triage to avoid wasting expensive and limited resources like VAD or cardiac transplant. Further studies are needed to evaluate the outcome of this bridging strategy.

## Electronic supplementary material

Below is the link to the electronic supplementary material.

Supplementary Material 1

## Data Availability

Data available on request. The data underlying this article will be shared on reasonable request to the corresponding author.

## Abbreviations

BIVAD: Biventricular Assist Device
BMI: Body Mass Index
cECMO: Central Extracorporeal Membrane of Oxygenation
CM: Cardiomyopathy
DCM: Dilated Cardiomyopathy
ECMO: Extra Corporeal Membrane Oxygenation
ICU: Intensive Care Unit
LVAD: Left Ventricular Assist Device
MCS: Mechanical Circulatory Support
MV: Mechanical Ventilation
pECMO: Peripheral Extracorporeal Membrane of Oxygenation
RRT: Renal Replacement Therapy
SAPS-II: Simplified Acute Physiology Score II
SOFA: Sequential Organ Failure Assessment
STROBE: Strengthening the Reporting of Observational Studies in Epidemiology
TAH: Total Artificial Heart
VAD: Ventricular Assist Device
