# Supplementary material for: Peripheral-to-central extracorporeal corporeal membrane oxygenation switch in refractory cardiogenic shock patients: outcomes and bridging strategies
Source: Ann Intensive Care. 2024 Oct 7;14:154. doi: 10.1186/s13613-024-01382-3 (PMC11458847; doi:10.1186/s13613-024-01382-3)
Supplement: Supplementary file 1 — Supplementary Material 1 [file 13613_2024_1382_MOESM1_ESM.docx]

| **Supplemental Table 1. Missing Data** | | |
| --- | --- | --- |
| **Variable** | **Missings (n)** | **Percent (%)** |
| Female | 0 | 0.0 |
| Age | 0 | 0.0 |
| Body mass index | 0 | 0.0 |
| Date of ICU | 0 | 0.0 |
| Arterial hypertension | 0 | 0.0 |
| Obesity | 0 | 0.0 |
| Chronic Cardiopathy | 0 | 0.0 |
| Smoker | 0 | 0.0 |
| Diabetes | 0 | 0.0 |
| Immunodepression | 0 | 0.0 |
| Cardiogenic shock etiology | 0 | 0.0 |
| Cardiac arrest before first MCS | 0 | 0.0 |
| SAPSII ICU | 3 | 3.8 |
| SOFA Total ICU | 4 | 5.0 |
| Charlson ICU | 3 | 3.8 |
| Peripheral va-ECMO | 0 | 0.0 |
| Impella | 0 | 0.0 |
| Intraaortic conterpulsation | 0 | 0.0 |
| ICU – pMCS Delay | 0 | 0.0 |
| Centralization reasons > 1 | 0 | 0.0 |
| Refractory pulmonary edema | 0 | 0.0 |
| Circulatory insufficiency | 0 | 0.0 |
| Cannula site infection | 0 | 0.0 |
| Lower limb ischemia | 0 | 0.0 |
| Peripheral MCS complications | 0 | 0.0 |
| Cardiac or aortic clotting | 0 | 0.0 |
| ICU – cMCS Delay | 0 | 0.0 |
| pMCS – cMCS Delay | 0 | 0.0 |
| Amines cMCS Delay | 0 | 0.0 |
| RRT at centralization | 0 | 0.0 |
| IMV duration before centralization | 0 | 0.0 |
| IMV > 48H before centralization | 0 | 0.0 |
| IMV Duration | 0 | 0.0 |
| Sepsis at cMCS | 0 | 0.0 |
| ATB at cMCS | 0 | 0.0 |
| Sepsis in H48 cMCS | 0 | 0.0 |
| pECMO – cECMO delay | 0 | 0.0 |
| Platelets at centralization | 4 | 5.0 |
| Fibrinogen at centralization | 5 | 6.2 |
| PT ratio at centralization | 5 | 6.2 |
| Bilirubin at centralization | 4 | 5.0 |
| SOFA Total at centralization | 3 | 3.8 |

| **Supplemental Table 2. Univariate And Multivariate Cox Model Analysis Of Pre-cECMO Factors Associated with One-Year Mortality** | | | | |
| --- | --- | --- | --- | --- |
| **Variables** | **Univariable** | | **Multivariable** | |
|  | **HR (95% CI)*^1^*** | **p-value** | **HR (95% CI)*^1^*** | **p-value** |
| Female | 0.76 (0.44 to 1.30) | 0.3 |  |  |
| Age, years | 1.01 (0.99 to 1.03) | 0.2 |  |  |
| Body mass index, kg/m2 | 1.04 (1.00 to 1.09) | 0.07 |  |  |
| Study period |  |  |  |  |
| 2006-2011 | — |  |  |  |
| 2012-2017 | 1.11 (0.62 to 1.98) | 0.7 |  |  |
| 2018-2023 | 1.01 (0.49 to 2.07) | 0.9 |  |  |
| ICU admission SAPS II score | 1.01 (1.00 to 1.03) | 0.2 |  |  |
| ICU admission SOFA score | 1.04 (0.98 to 1.10) | 0.2 |  |  |
| Charlson comorbidity index | 1.23 (1.00 to 1.51) | 0.054 |  |  |
| ICU to pECMO time, days | 0.97 (0.91 to 1.03) | 0.3 |  |  |
| ICU to cECMO time, days | 0.99 (0.97 to 1.02) | 0.5 |  |  |
| pECMO to cECMO time, days | 1.00 (0.98 to 1.02) | >0.99 |  |  |
| Past medical history |  |  |  |  |
| Arterial hypertension | 1.13 (0.60 to 2.14) | 0.7 |  |  |
| Obesity | 1.74 (0.95 to 3.20) | 0.07 |  |  |
| Cardiomyopathy | 1.09 (0.59 to 2.02) | 0.8 |  |  |
| Diabetes | 1.30 (0.52 to 3.26) | 0.6 |  |  |
| Immunocompromised | 0.81 (0.32 to 2.02) | 0.6 |  |  |
| Cause of cardiogenic shock |  |  |  |  |
| Myocarditis | — |  | — |  |
| Myocardial infarction | 2.40 (1.26 to 4.58) | 0.008 | 2.53 (1.28 to 5.01) | 0.008 |
| Dilated cardiomyopathy | 1.39 (0.67 to 2.88) | 0.4 | 1.62 (0.76 to 3.45) | 0.2 |
| Others | 0.57 (0.17 to 1.97) | 0.4 | 0.57 (0.13 to 2.58) | 0.5 |
| First MCS |  |  |  |  |
| Cardiac arrest before first MCS | 0.98 (0.55 to 1.72) | 0.9 |  |  |
| IMPELLA® | 1.28 (0.55 to 2.98) | 0.6 |  |  |
| Intraaortic conterpulsation | 1.56 (0.93 to 2.62) | 0.09 |  |  |
| Reason for cECMO switch |  |  |  |  |
| >1 reason | 1.06 (0.63 to 1.77) | 0.8 |  |  |
| Pulmonary edema | 0.95 (0.56 to 1.61) | 0.8 |  |  |
| Circulatory failure | 1.04 (0.61 to 1.78) | 0.9 |  |  |
| Cannulation site infection | 0.99 (0.51 to 1.91) | 0.9 |  |  |
| Lower limb ischemia | 1.77 (0.64 to 4.89) | 0.3 |  |  |
| Cardiac cavities pre-thrombotic state | 1.22 (0.63 to 2.35) | 0.6 |  |  |
| Organ failures before cECMO |  |  |  |  |
| RRT at centralization | 1.80 (1.06 to 3.05) | 0.03 | 1.27 (0.56 to 2.90) | 0.6 |
| MV duration before centralization | 1.01 (0.99 to 1.04) | 0.3 |  |  |
| Time on MV before cECMO, days | 0.99 (0.98 to 1.00) | 0.03 |  |  |
| cECMO-day parameters |  |  |  |  |
| SOFA score | 1.10 (1.01 to 1.20) | 0.03 | 1.05 (0.92 to 1.19) | 0.5 |
| Platelets, G/L | 1.00 (0.99 to 1.00) | 0.6 |  |  |
| Fibrinogen, g/L | 0.89 (0.77 to 1.01) | 0.08 |  |  |
| Prothrombin time ratio, % | 0.99 (0.97 to 1.00) | 0.1 |  |  |
| Bilirubin, IU/L | 1.00 (1.00 to 1.00) | 0.9 |  |  |
| Abbreviations: HR, Hazard Ratio; CI, Confidence Interval; cECMO, central Extracorporeal Membrane Oxygenation; ICU, Intensive Care Unit; MCS, Mechanical Circulatory Support; MV, Mechanical Ventilation, SAPS-II, simplified acute physiology score II; SOFA, Sequential Organ Failure Assessment | | | | |

| Supplemental Table 3. General and cECMO Characteristics, Complication And Outcome In The 51 Patients With Bicentrifugal Biventricular Support | | | | |
| --- | --- | --- | --- | --- |
| Variables | **All patients n=51** | **Hospital discharge** | | **p-value** |
|  |  | **Survivors n=15** | **Non-survivors**  **n=36** |  |
| Female | 13 (25) | 3 (20) | 10 (28) | 0.7 |
| Age, years | 44±13 | 43±13 | 44±14 | 0.9 |
| Body mass index, kg/m^2^ | 26.4±5.4 | 24.0±5.8 | 27.5±5.0 | 0.03 |
| Day-0 SAPS II score | 61 [43-72] | 57 [37-68] | 62 [47-72] | 0.2 |
| Day-0 SOFA score | 12 [8-14] | 9 [8-14] | 12 [9-14] | 0.2 |
| Charlson comorbidity index | 1 [1-2] | 1 [1-3] | 1 [1-2] | 0.5 |
| Past medical history |  |  |  |  |
| Obesity | 13 (25) | 1 (67) | 12 (33) | 0.08 |
| Arterial hypertension | 12 (24) | 3 (20) | 9 (25) | >0.99 |
| Cardiomyopathy | 11 (22) | 6 (40) | 5 (14) | 0.06 |
| Diabetes | 5 (10) | 1 (7) | 4 (11) | >0.99 |
| Immunocompromised | 5 (10) | 2 (13) | 3 (8) | 0.6 |
| Cause of the cardiogenic shock |  |  |  | 0.1 |
| Myocardial infarction | 22 (43) | 4 (27) | 18 (50) |  |
| Myocarditis | 13 (25) | 4 (27) | 9 (25) |  |
| Dilated cardiomyopathy | 12 (24) | 4 (27) | 8 (22) |  |
| Others^a^ | 4 (8) | 3 (20) | 1 (3) |  |
| First MCS |  |  |  |  |
| Cardiac arrest before first MCS | 20 (39) | 5 (33) | 15 (42) | 0.6 |
| Peripheral ECMO | 51 (100) | 15 (100) | 36 (100) | na |
| IMPELLA® | 6 (12) | 1 (7) | 5 (14) | 0.7 |
| Intraaortic balloon pump | 27 (53) | 7 (47) | 20 (56) | 0.6 |
| Organ failures before cECMO |  |  |  |  |
| Renal replacement therapy | 29 (57) | 5 (33) | 24 (67) | 0.03 |
| Mechanical ventilation >48h | 39 (76) | 9 (60) | 30 (83) | 0.1 |
| Time on MV, days | 4 [2-17] | 2 [1-10] | 10 [2-20] | 0.02 |
| Parameters on cECMO-day |  |  |  |  |
| SOFA score | 17 [15-20] | 16 [15-18] | 18 [16-20] | 0.07 |
| Platelets, G/L | 86 [47-144] | 111 [60-138] | 80 [45-147] | 0.4 |
| Fibrinogen, g/L | 4.6 [2.4-6.2] | 4.9 [3.5-6.2] | 4.6 [2.2-6.2] | 0.5 |
| Prothrombin time, % | 59 [48-69] | 62 [51-71] | 56 [48-66] | 0.4 |
| Bilirubin, UI/L | 39 [18-62] | 23 [16-69] | 41 [20-61] | 0.4 |
| Complication under cECMO |  |  |  |  |
| Renal replacement therapy | 41 (80) | 9 (60) | 32 (89) | 0.05 |
| Hemothorax or tamponade | 26 (51) | 5 (33) | 21 (58) | 0.2 |
| Surgical revision | 18 (35) | 5 (33) | 13 (36) | 0.8 |
| Time from cECMO to first revision, days | 3 [0-12] | 3 [0-3] | 7 [0-16] | 0.3 |
| Mediastinitis | 18 (35) | 9 (60) | 9 (25) | 0.02 |
| Time from cECMO to mediastinitis, days | 14 [10-30] | 30 [24-46] | 11 [8-14] | 0.03 |
| Stroke | 17 (33) | 3 (20) | 14 (39) | 0.2 |
| cECMO thrombosis | 0 (0) | 0 (0) | 0 (0) | na |
| Outcomes |  |  |  |  |
| Time from ICU admission to cECMO, days | 13 [5-25] | 13 [5-21] | 13 [5-25] | 0.8 |
| Time from first MCS to cECMO, days | 11 [4-21] | 9 [4-14] | 12 [4-22] | 0.35 |
| Time on MCS, days | 16 [9-37] | 30 [12-42] | 15 [3-25] | 0.04 |
| Time on cECMO, days | 16 [9-37] | 30 [12-42] | 15 [3-24] | 0.06 |
| Time in ICU, days | 40 [27-64] | 65 [44-92] | 33 [18-52] | <0.001 |
| Time in hospital, days | 45 [29-67] | 84 [57-107] | 36 [19-53] | <0.001 |
| Bridging strategies, n=30 |  |  |  | 0.007 |
| Bridge-to-recovery | 4 (24) | 4 (27) | 0 (0) |  |
| Bridge-to-transplantation | 10 (59) | 10 (67) | 0 (0) |  |
| Bridge-to-VAD |  |  |  |  |
| LVAD | 2 (12) | 0 (0) | 2 (100) |  |
| TAH | 1 (6) | 1 (7) | 0 (0) |  |
| Successful cECMO weaning | 19 (37) | 15 (100) | 4 (11) | <0.001 |
| Day-90 mortality | 35 (69) | 0 (0) | 35 (97) | <0.001 |
| One-year mortality | 38 (75) | 2 (13) | 36 (100) | <0.001 |
| Continuous variables are expressed as mean ± standard deviation or median [interquartile range 25-75] and compared with Student’s t-test or Wilcoxon’s rank test; categorical variables are expressed as n (%) and compared with Fischer’s exact test.  Abbreviations: cECMO, central Extracorporeal Membrane Oxygenation; ICU, Intensive Care Unit; LVAD, Left Ventricular Assist Device; MCS, Mechanical Circulatory Support; MV, Mechanical Ventilation; SAPS-II, simplified acute physiology score II; SOFA, Sequential Organ Failure Assessment; TAH, Total Artificial Heart; ECMO, Extracorporeal Membrane of Oxygenation; VAD, Ventricular Assist Device. ^a^Hypertrophic cardiomyopathies n=2, catastrophic antiphospholipid syndrome n=1, iatrogenic rupture of tricuspid papillary muscle n=1. | | | | |

| **Supplemental Table 4. Key Variables Across Study Periods** | | | | |
| --- | --- | --- | --- | --- |
| **Variables** | **2006-2011**  **n=42** | **2012-2017**  **n=24** | **2018-2023**  **n=14** | **p-value*^1^*** |
| Age, years | 42 [27-51] | 51 [38-56] | 39 [22-50] | 0.04 |
| Female | 20 (48) | 6 (25) | 4 (29) | 0.1 |
| ICU admission SAPS-II score | 65 [56-73] | 55 [36-71] | 59 [48-65] | 0.07 |
| Cause of cardiogenic shock |  |  |  | 0.7 |
| Myocarditis | 14 (33) | 5 (21) | 6 (43) |  |
| Ischemic cardiomyopathy | 15 (36) | 11 (46) | 4 (29) |  |
| Dilated cardiomyopathy | 11 (26) | 5 (21) | 3 (21) |  |
| Other | 2 (5) | 3 (13) | 1 (7) |  |
| Intraaortic balloon pump | 11 (26) | 10 (42) | 12 (86) | <0.001 |
| Main cause of centralization |  |  |  | 0.2 |
| Cannula infection | 1 (2) | 3 (13) | 0 (0) |  |
| Cardiac or aortic clotting | 2 (5) | 1 (4) | 4 (29) |  |
| Circulatory insufficiency | 10 (24) | 4 (17) | 1 (7) |  |
| Limb ischemia | 3 (7) | 1 (4) | 1 (7) |  |
| Pulmonary edema | 26 (62) | 15 (63) | 8 (57) |  |
| In-hospital mortality | 28 (67) | 17 (71) | 10 (71) | 0.9 |
| One-year mortality | 29 (69) | 19 (79) | 10 (71) | 0.7 |
| Continuous variables are expressed as mean ± standard deviation or median [interquartile range 25-75], categorical variables are expressed as n (%) and compared Kruskal-Wallis rank sum test, Pearson’s Chi-squared test or Fisher’s exact test when appropriate. Abbreviations: SAPS-II, simplified acute physiology score II. | | | | |

**Supplemental Figure 1. Number Of Cannulas Across Study Periods**

**
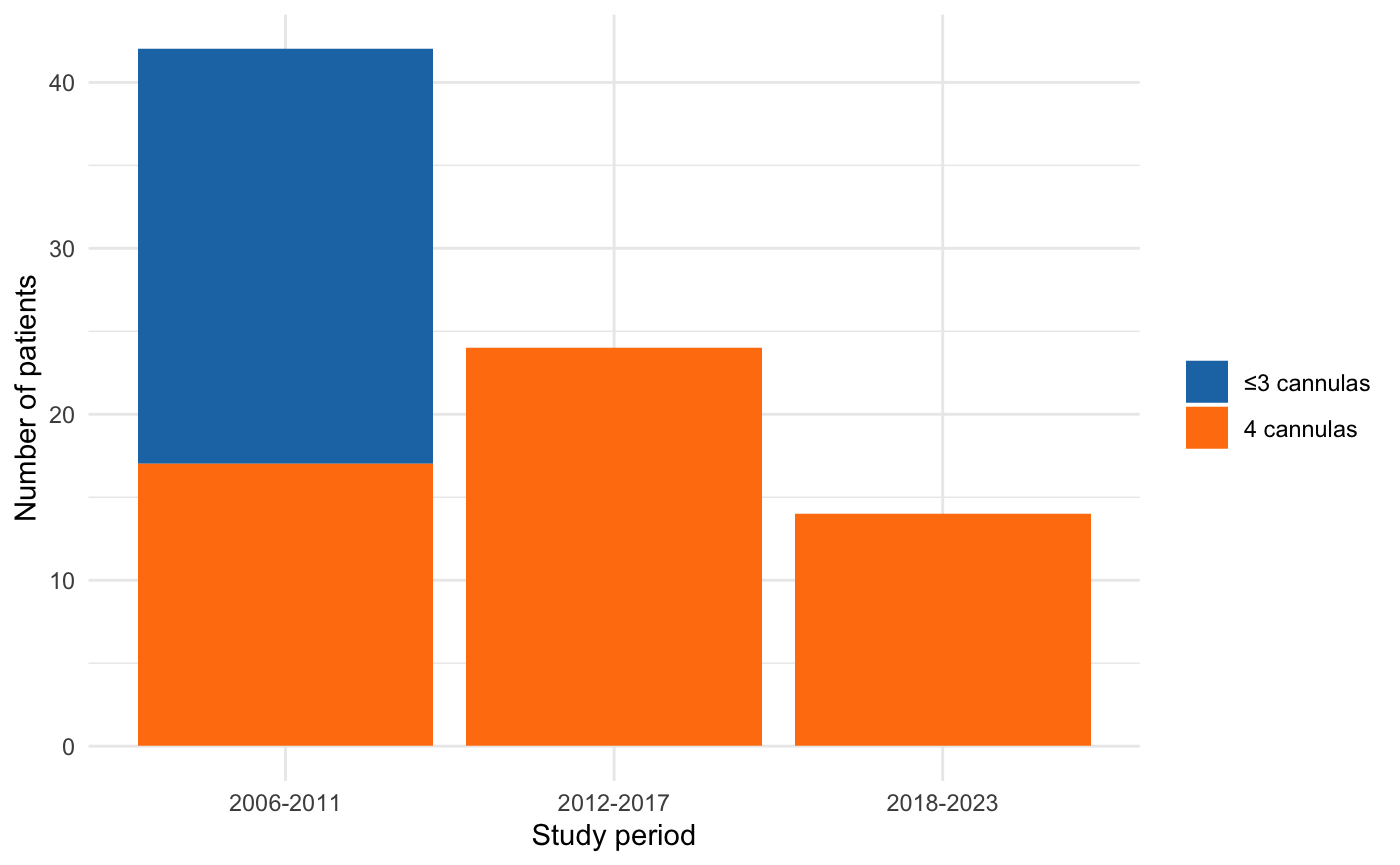
**

**Supplemental Figure 2. Causes Of Centralization Across Study Periods**

**
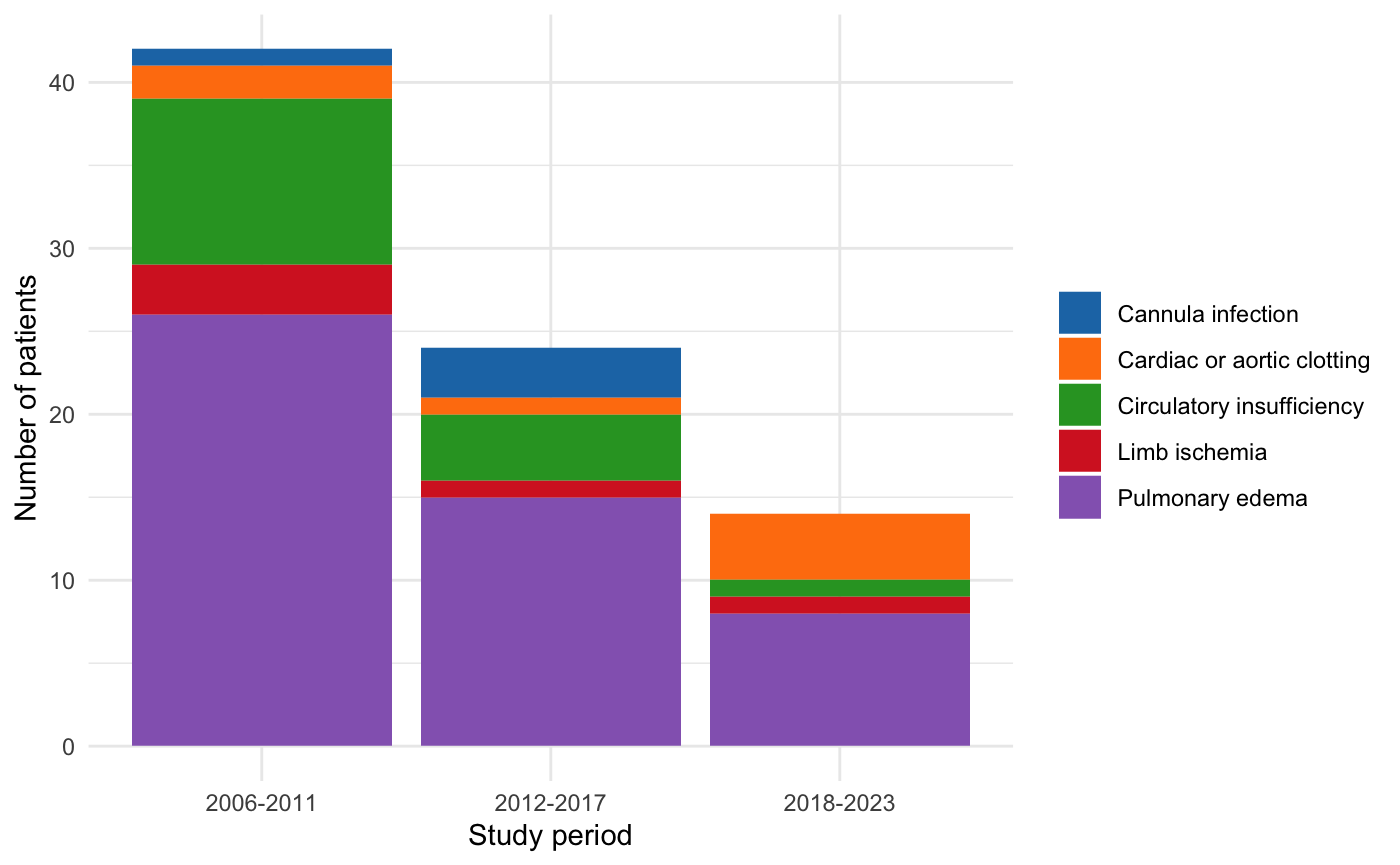
**
